# An Impact Mapping Method to Generate Robust Qualitative Evaluation of Community-Based Research Programs for Youth and Adults

**DOI:** 10.3390/mps1030025

**Published:** 2018-07-17

**Authors:** Melissa D. Olfert, Rebecca L. Hagedorn, Jade A. White, Barbara A. Baker, Sarah E. Colby, Lisa Franzen-Castle, Kendra K. Kattelmann, Adrienne A. White

**Affiliations:** 1 Division of Animal and Nutritional Sciences, Davis College of Agriculture, Natural Resources & Design, West Virginia University, G025 Agricultural Science Building, Morgantown, WV 26506, USA; rlhagedorn@mix.wvu.edu (R.L.H.); jade_white@my.uri.edu (J.A.W.); 2 4-H Youth Development, University of Maine Cooperative Extension, 307 Maine Avenue, Bangor, ME 04410, USA; barbara.baker@maine.edu; 3 Department of Nutrition, University of Tennessee, 1215 W. Cumberland Avenue, 229 Jessie Harris Building, Knoxville, TN 37996-1920, USA; scolby1@utk.edu; 4 Department of Nutrition and Health Sciences, University of Nebraska-Lincoln, 110 Ruth Leverton Hall, Lincoln, NE 68583-0806, USA; lfranzen2@unl.edu; 5 Department of Health and Nutritional Sciences, South Dakota State University, Box 2203, SWG 443, Brookings, SD 57007, USA; kendra.kattelmann@sdstate.edu; 6 School of Food and Agriculture, University of Maine, 5735 Hitchner Hall, Orono, ME 04469, USA; awhite@maine.edu

**Keywords:** ripple effect mapping, youth, adult, methods, impact mapping, implementation and dissemination, community, sustainability

## Abstract

Ripple Effect Mapping (REM) is an evaluation approach that has traditionally been used in community settings to visually map the impact of programming and community interventions. This manuscript utilizes the Community Capitals Framework (CCF) to inform REM and to better highlight the changes and impact between various levels of a community, following a childhood obesity prevention intervention. The addition of in-depth qualitative analyses makes this approach particularly useful for the evaluation of interventions with a research–community partnership focus. The objective of this study was to describe a CCF-informed REM approach with detailed protocol, training, and application to the community-based, childhood obesity prevention intervention, iCook 4-H, which targeted youth and adult pairs. This protocol includes the steps required to prepare for REM sessions of, ideally, six youth and adult pairs, one facilitator, and one or two evaluators/note takers. REM sessions typically begin with an icebreaker and appreciative inquiry activities that inform the REM mapping process that follows. In-depth qualitative analysis of the notes and map images captured during REM sessions ensure the rigor required for research-related interventions. Researchers, community members, and participants can use CCF-informed REM collectively as a robust evaluation tool to demonstrate, through visual mapping, the positive effects of community-partnered research programs.

## 1. Introduction

Research to examine effective implementation and dissemination of community programming has increased within the last 25 years [1,2]. Subsequently, there has been an increased need for methods to systematically evaluate the impact of implemented and disseminated programming. These methods can help in bridging the research to practice gap [1,3,4]. To understand the reach and impact of evidenced-based programs within communities, stepwise tools that evaluate these programs are needed [5]. Evaluations of the impact of programs can include increased motivation within the community, strengthened community member involvement, positive lifestyle alterations, and increased program funding resulting in short-term sustainability [2,6]. This has led to the publication of many tools and frameworks to measure impact evaluation within community programming with hopes of improving implementation of evidence-based practice into non-research settings [7,8,9,10,11].

Impact evaluation is a process that enables researchers, participants, and community members to understand the efficacy and reach of programming on its targeted population. Furthermore, this evaluation information can improve future programming [12]. Ripple Effect Mapping (REM) is one type of impact evaluation that assesses how effectively programs reach their goals [13]. The adoption of an evaluation tool such as REM can assist in producing evidence of positive community program outcomes. REM documents various impacts, positive outcomes, and changes that result from a particular program through using the Community Capitals Framework (CCF) [14,15,16]. CCF was created to better analyze how successful communities work and to serve as a platform to determine the success of other communities and their programs [14,15]. Sustainable and entrepreneurial communities possess seven areas of strength and resourcefulness that are interrelated and combined to promote economic development and viable community enhancements [14,15,17]. These seven areas are known as ‘capitals’ (Table 1) or resources that the community uses to build upon and form new assets [14,15]. In REM evaluations, CCF is used to document programmatic impacts on participants, stakeholders, and the surrounding community [14,15]. Thus, during REM using CCF, program participants are invited to reflect on what parts of a community have been changed by their action(s) (who and how) and how it affected the way institutions in that community “do business” on a daily basis. The three REM questions juxtaposed within the seven Community Capitals add different dimensions to the answers, and show not only personal value but also encompass broader issues backed by research-based facts. This evaluation tool has been found to be effective when compared to previously used evaluation tools, such as outcome mapping and the Most Significant Change method, in analyzing experimental research to yield more useful or detailed information [18,19]. Therefore, REM with CCF can help community and program stakeholders determine positive program outcomes within the community health promotion sector [20].

Although REM with CCF is a proposed impact evaluation method, it has not been widely adopted within the research sector for health promotion programming. REM has been used in Extension programming to evaluate projects including the Horizons leadership program [21], the “Tide” program for civil dialog on poverty [22], and community development [14]. Further, when used in research, the data analysis is commonly limited to coding the community capitals or open coding and lacks a strong qualitative analysis [19]. To facilitate the translation of research into evidenced-based practice, a stepwise process must be described to be used in future research and successfully translate findings into useable and understandable information to the participants, community, and larger public [1]. The lack of investigation in impact evaluation tools for community interventions that promote sustainability can begin to be remedied by utilizing existing tools that contribute to the need for disseminated, evidenced-based programs within the community sector [4].

Specifically, because of increasing rates of obesity in children, many prevention programs have been developed in the past decade to alleviate this trend [23]. Childhood obesity prevention researchers have applied various methods to reduce obesity including physical activity, culinary skills, and/or healthier food choices, with different levels of success [24]. In addition, theoretical foundations of these programs often vary [25]. One commonly used theoretical foundation for childhood obesity programs is social cognitive theory (SCT), in which children can achieve behavior change through increased self-regulation or self-efficacy [25,26,27]. In addition, many programs involve parental involvement, which can further build on SCT constructs in obesity prevention program [28]. However, while many of these programs are successful during their intervention phase, after research intervention ends, the sustainability and longevity of these programs in the community are lacking or unknown [24,29].

Following the two-year iCook 4-H obesity prevention program [30], REM sessions occurred to assess program impact, and the methodology is presented here. Although there have been many efforts for program evaluation, some which use community mapping, to our knowledge, there is no comprehensive description of a stepwise, detailed methodology of a REM evaluation tool with CCF incorporating content analysis and word frequencies for a community based, childhood-obesity program, as was used for the iCook 4-H program. The objective of this article was to describe REM methodology to evaluate community-based, obesity prevention research programs, with results presented elsewhere.

## 2. Methodology

The REM process takes approximately 90–120 min to complete and includes a structured group activity (similar to a focus group) with sequential questioning designed to elicit reflection and feedback from participants. A map, a visual picture of responses, is created by a trained facilitator as the discussion ensues. The facilitator should be familiar with the research program and participants, preferably the individual who led the community intervention and with whom the participants are familiar. Trained note taker(s) record session discussions and input data into a report template. These note-takers can be researchers or other community members trained in the process. The following sections detail the stepwise components necessary for completing a REM session in a community-based research program.

### 2.1. Pre- Ripple Effect Mapping Session

#### 2.1.1. Training

Training videos on the REM process were developed and are available online [31]. Specific for REM with youth and adults, a written training toolkit was created and is available as a supplement to this methodology report. Training and guides are helpful to ensure homogeneity across REM sessions for optimal data comparison. Each note-taker should also be trained in using a report template so that both youth and adult comments can be captured along with using CCF. Note-takers can vary between REM sessions and can be trained by the facilitator to record statements and to denote whether statements came from youth or adults.

#### 2.1.2. Participants

Participants that completed the research program should be invited to participate in REM session. Focus groups include both youth and adult participants together and should contain a minimum of 3 youth and 3 adult participants and no more than 6 of each per session (12 total people), to stay within recommended limits for focus group data collection [32]. Having too many participants can limit the opportunity for participants to share their insights and change group dynamics. For comprehensive program evaluation, more than one session should occur to reach saturation of feedback.

#### 2.1.3. Required materials

**Figure 1:**
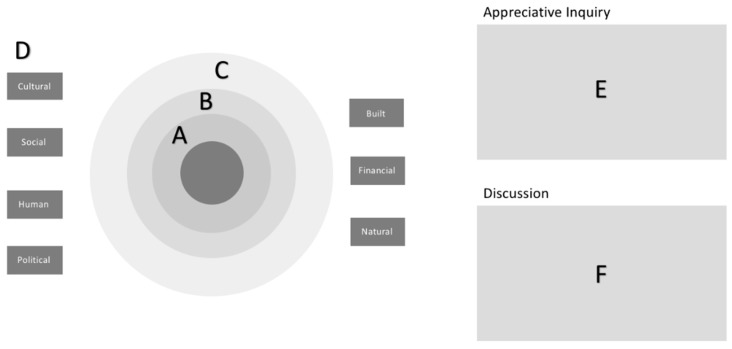
Ripple Effect Mapping (REM) area components. Sample mapping components set up. A—ring for mapping questions 1; B—ring for mapping question 2; C—ring for mapping question 3; D—community capitals posted around map; E—area beside map for Appreciative Inquiry; F—area beside map for discussion. Further details provided in Section 2.2.

For the REM session, the following materials are required. A whiteboard for constructing the map, which could also be a variation of a whiteboard (such as a butcher paper, chalkboard, larger poster board, or pieces of paper taped together to fit a large area). Next, four different colored markers are needed, as each “ripple” of the map will be indicated by a different color. For participants, pens and paper can be provided to allow for note taking. A short demographic survey (including gender, age, ethnicity, etc.) is needed as well to capture sample population characteristics. Additionally, a laptop and audio recorder are highly encouraged, but not required to capture data. The laptop will be used by the note-taker during the REM session with the audio recorder capturing any responses the note-taker might have missed, particularly connecting comments to youth and adults. Further, for data capturing, a camera (it can be digital or cell phone camera) is required to take a photo of the map at the end of the REM session, ideally with all participants and facilitators around it (optional) and especially of the map alone with a close up to be able to read the notes. Lastly, participant handouts and printouts of the community capitals are necessary. Depending on the organization/institution, consent forms and media releases may be required by institutional review boards (IRB) or governing bodies and should be considered. Additionally, incentivizing can be used for participation and should be reviewed with the appropriate organization/institution oversight committee. A sample of posted mapping components is seen in Figure 1.

### 2.2. Ripple Effect Mapping Session

#### 2.2.1. Setting and Introduction

The layout of the room used for REM activity is very important for creating an effective facilitator-led discussion. A horseshoe or half-circle seating arrangement is recommended to optimize participant interaction. Begin REM sessions with introductions by the facilitator and the note-taker, describe REM session participants by having them complete a brief demographic survey. Guidelines for group discussions should be reviewed to ensure a respectful environment during REM sessions. Basic guidelines include being polite when other participants are speaking, allowing youth participants to speak before adults, and respecting other participants’ privacy once the session is over. Group dynamics, such as participant placement around the sitting area, should be recorded by the note-taker.

#### 2.2.2. Icebreaker and Appreciative Inquiry Activities

The facilitator leads an icebreaker activity to allow participants to become comfortable with the group and stimulate open communication. It is recommended that the ice breaker activity be associated with the research program in which the subjects participated. For example, an activity could consist of a question and answer matching game based on information included in the program. Following the icebreaker activity, the facilitator leads an appreciative inquiry activity using a separate paper on one side of the map for the purpose of participants identifying the most beneficial program aspects. Appreciative Inquiry is a method used to help enterprises become more effective by adopting an overall way of thinking that focuses on solutions and positives, with the belief being that focusing on positives will in turn create more solutions and positives [33,34]. In general, individuals talk about parts of the program that they care about and act upon [35]. By using AI, REM focuses on positive program aspects to determine which beneficial changes were made, providing a second theoretical construct for the analysis. When both youth and adults take part, conversational comfort level is typically higher when each pairs with a like-aged participant [14]. These participants are instructed to interview one another about favorite memories from the research program. The facilitator records these topics called “Action Statements” on the side of each map. Participants refer to Action Statements in dialogue throughout the session to build a story thread from action to value for individuals and the community as they answer the three questions showing the action’s ripple effect.

#### 2.2.3. Introduce REM Concepts

Prior to beginning the REM mapping, the facilitator should explain why the session is being conducted and what participants can expect within the 90–120-min session. A sample explanation is

“This session will give us a better understanding of your experience with the [insert research program here] and how it impacted you individually and as a group and/or community. This map will be a tool for us to understand the effects this program has had on your families and in the community through a ‘rippling effect.’ This ‘ripple effect’ is like a pebble being dropped into a pond; one small pebble can make a large impact of ripples throughout a pond. Similarly, a singular program can make small and large impacts within a family and community by individuals passing on their new experiences and knowledge learned from the program to others.”

Next, the facilitator should provide an explanation of the community capitals that will be used throughout the session. Descriptions of each capital can be found in Table 1. These definitions can be modified by the facilitator to provide youth-friendly terms in place of REM and community capitals, which can assist in the clarity of instruction. However, loss of the CCF theoretical framework may affect comparison studies. Participants should be given a handout of the capitals for reference during the session. The community capitals are a way in which participants can relate positive impacts of the research program to affected resources within the community.

#### 2.2.4. Mapping Process

**Figure 2:**
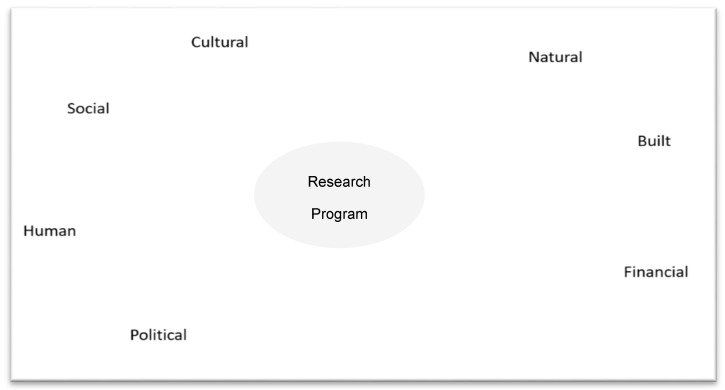
Sample mapping template.

The mapping process is completed in accordance with the “Ripple Effect Mapping Field Guide [19]” and provided here for use with youth and adult participants. The facilitator starts by placing the program title in a central area on board media to start map. A sample map is represented in Figure 2.

The facilitator, through discussion with participants, should apply three to four broad themes from the appreciative inquiry activity around the research title in the middle of the map. For example, if participants completed a culinary program, they might state their favorite memories were “developing cooking skills”, “eating healthy foods”, and “family time physical activity”, as in the iCook 4-H program [30,36]. These three themes are the focus for the mapping questions. The mapping process occurs with three opened-ended questions for each theme, in which youth and adults respond to leader-facilitated dialogue about ways in which the program impacted the individual, their family unit, and/or their community. The facilitator must explain to the group that it is encouraged for youth participants to comment first on each question, with the adults commenting after youth ideas are stated.

The first question is “What are people doing differently as a result of *[insert research program action statement here]*? Allow time for each participant to comment and each note taker to capture responses. Both the facilitator and note taker should document whether the response was a youth or adult with a marked “Y” or “A” following the comment. Allow participants time to correct, clarify, or add to what the facilitator writes on the map to ensure credibility of the data. Repeat the question for each of the 3–4 important themes reported. Once every participant has had the opportunity to comment, the facilitator should then ask the group what capital is related to each comment. A line will be drawn to each capital and comment in the same color marker. Each comment should have a square next to it with the letter of the capital inside as well (i.e., N = Natural, C = Cultural, H = Human, S = Social, P = political, F = Financial, B = Built).

Following the same guidelines, the next two questions can be asked and a different colored marker can be used to indicate responses for each question. The second question is “Who has benefited from *[insert research program here]* and how?”, and the third question is “Are there changes in the way community groups and institutions do things as a result of *[insert research program here]*? Remember to continue to document whether an adult or youth made the comment and what capital is related to which comment within the square for each. Through this process, the facilitator documents the participants’ thoughts on how one event or outcome affected another; this is termed mapping the “ripples” that have taken place [5,20].

**Figure 3:**
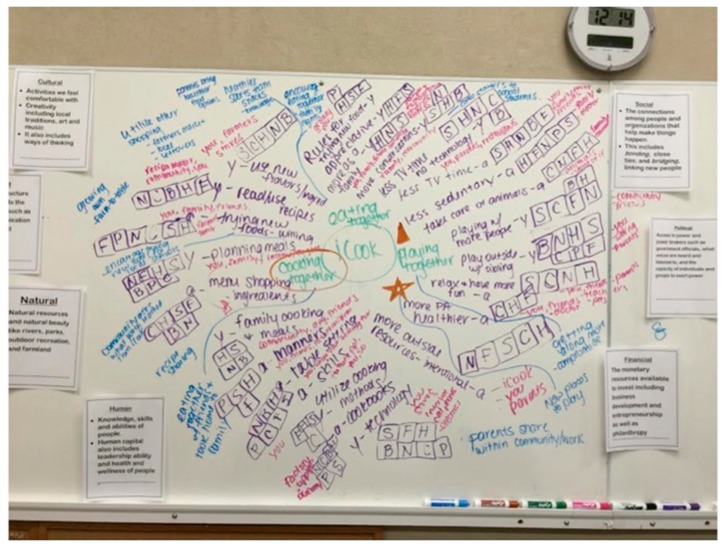
Sample finished REM map from iCook 4-H.

Following the questions and community capitals discussion, the facilitator should discuss with participants the most important or significant idea, most bridging among new people, and most bonding among the group/participants that emerged from the mapping process. Participants can discuss among themselves, noting the most accepted response. If there is a discrepancy between the participants, the note-taker should note the other ideas brought up during discussion. To represent the most important or significant impact on the map, the facilitator should label beside the comment with a star. Following the same process, a circle is placed on the map to represent the most bonding for bringing the group or family together, and a square represents the most bridging to new people. This activity reflects the importance of social capital within a community, giving participants additional time to reflect on program events that lead to positive community cohesion. A sample, finished map is represented in Figure 3.

### 2.3. Post Session

The facilitator or note-taker should capture a photographic image of the REM map following the session. The photograph can be taken with REM participants if they choose, but a participant-free photo should also be captured for reference in analysis. Facilitators and note-takers should converse immediately following the REM session to document any additional limitations perceived to have occurred during the sessions and what the facilitator perceived as having worked best during the process. Further, member checking, otherwise termed as expert reviewer debriefing, should occur to ensure an accurate depiction of participant perceptions from the REM session. The data should be entered into a data report template (Supplemental file) for data analysis, with use of the map picture to transcribe all data.

## 3. Analysis

Inductive content analysis is recommended since a dyad approach in a research setting is weakly discussed in the literature [37]. This process includes open coding, category creation, and abstraction. To begin, use the data report templates completed by note takers from the REM session. For open coding, the primary researcher should study the data line by line, first making general field notes and headings [38]. A second pass of the report template is suggested to certify no other codes were missed. For this specific analysis, care should be taken to note if youth or adults are making the comments, with different coding for age as appropriate. Once codes are complete, categories can be created by merging codes into higher order headers to create categories, with the goal of placing data into like groups for comparison with other groups [39,40,41]. The data should be abstracted further into subcategories and sub-subcategories, as far as the researcher sees possible [39]. For reliability, two additional researchers should review each report template and the theme, categories, subcategories, and supporting subcategories. Further, words related to the categories can be counted for word frequencies and used to show word clouds as visual representations of the most common words from the session. The rigor of data analysis should be gained though credibility, applicability, consistency, and neutrality during the process [42].

## 4. Discussion

Adopting a framework to evaluate the effect of interventions and translation of sustainable action is needed to deploy with interventions addressing preventive measures for widespread chronic disease within the community setting [43,44]. Frameworks addressing the implementation and dissemination need to provide summaries of phases outlining quality implementation [1]. Thus, REM can act as a feedback mechanism and evaluation tool, providing impact of program outcomes and participant perceptions. Its utilization of CCF helps participants and researchers see broader implications and impacts [14]. Additionally, it provides opportunities for researchers, community stakeholders, participants, and support staff to learn from these findings to make improvements in further dissemination. The comprehensive methodology of the CCF-informed REM evaluation tool presented here will aid future programs in using REM and CCF to evaluate programming.

Although REM has been used in various settings [19], including professional settings [45] and community programming [22], documentation of a stepwise REM process with detailed content analysis and word frequencies has not taken place, more specifically with both youth and adult participants. Thus, the innovative methodology presented here meaningfully contributes to the field of implementation science and be used in future research as an evaluation tool in childhood obesity prevention programs, including both youth and adults with qualitative analysis.

In context, REM is only one of many evaluation tools and techniques available to assess the impact of community research [1,4,7,9,10,46,47]. However, REM distinguishes itself here due to the inclusion of CCF. The use of a community capitals approach broadens the scope of the qualitative impact investigation to include various resources and relationships within the community and has the ability to evaluate change among each of the seven capitals within the community, as well as subsequent impacts across them [48]. Let us take an example, with a participant reporting: “Because I learned how to use recipes to cook, my brother began to like veggies and my family was inspired to contribute healthy dishes at events therefore the community members eating at the pot luck became healthier.” When applying CCF to this sample story thread, participants are able to identify that using recipes meant planning ahead with a shopping list (human capital). Perhaps one of the ingredients was not at first available in the local storeowner but was ordered and more than the one family began buying them (financial capital), thus increasing the bottom line of the store owner. Changing to include more healthy items in the pot luck recipes spurred change in the community healthy eating culture (human capital), not only with regard to healthy items, as more recipes from various cultures were incorporated too (cultural capital). People began using the vegetables grown in their community garden (natural capital) available at the food pantry and began to volunteer at the garden to make sure they continued to be available (political capital), urging continued community support of the gardens and food pantry. This example shows how a single REM participant response can be applied to multiple capitals within the community. This level of impact is lacking in previous evaluation methods, making REM a novel and powerful tool with which to evaluate program impact within a community [49].

While REM provides opportunity for program evaluation, it is not without limitations. First, REM largely focuses on positive outcomes, with participants’ reports of program failures, or program harms, less likely to be stated. Therefore, it is important that program failures are acknowledged and addressed before disseminating a program into the community. Next, with little researcher control over the data collected and the complexity of qualitative research, it is hard to distinguish causality [32]. While REM successfully captures participant perspectives, there are multiple views that need to be represented in program evaluations, including those of funders, staff and administrators, and participating organizations. Using REM with CCF in conjunction with larger evaluation frameworks that capture vast viewpoints, such as eB4CAST [50], could be beneficial. Lastly, qualitative research is a labor-intensive process that requires time commitment through the whole data collection and analysis process, which may be a barrier for some community programs [37].

## 5. Conclusions

This REM methodology has been presented in a way that had not been previously reported as a comprehensive, qualitative tool that could be used for robust evaluation following community-based programs. To understand program effects and outcomes, evaluation tools, such as REM, can be used to benefit participants, community members, and researchers by providing visual imaging or story boards through the ripple mapping of program impacts. Furthermore, using REM as a qualitative research evaluation tool gives a more telling and deeper story compared to quantitative findings only [51]. Therefore, applying a tool such as REM with CCF can be used by researchers, community members, and participants to demonstrate through a visual mapping image the positive effects of programs that seek to further disseminate and implement replicability and sustainably. Additional research is needed to verify the use of REM as an appropriate evaluation tool within community-based obesity prevention research to better support implementation and dissemination. Replicating this REM process using various evidenced-based programs within obesity prevention research can further document the effectiveness and impact of REM as an evaluation tool.

## Figures and Tables

**Table 1:** Community capital descriptions.

Social: Connections among individuals and groups that help make things happen, including bonding with people you know and bridging to new people or seeing people in unfamiliar roles.
Natural: Natural resources and natural beauty like rivers, parks, outdoor recreation, and farmland.
Cultural: Activities, foods, creativity (local traditions, art, and music), and ways of thinking that are familiar.
Human: Knowledge, skills, and abilities of people; also includes leadership ability and health and wellness of people.
Political: Access to decision-makers, such as student council, school boards, or town councils. Power of individuals and groups to influence rules or budgets.
Financial: Money available to invest, including helping or starting businesses, as well as giving away money and goods to those who need it.
Built: Structures and facilities that support a community, such as communications, roads, and buildings.

Descriptions of the seven community capitals found within the Community Capital Framework.
